# Multi-Agent Deep Reinforcement Learning for Collision-Free Posture Control of Multi-Manipulators in Shared Workspaces

**DOI:** 10.3390/s25226822

**Published:** 2025-11-07

**Authors:** Hoyeon Lee, Chenglong Luo, Hoeryong Jung

**Affiliations:** Department of Mechanical Engineering, Konkuk University, 120 Neungdong-ro, Gwangjin-gu, Seoul 05029, Republic of Korea; ehoyeon@konkuk.ac.kr (H.L.); luo0611@konkuk.ac.kr (C.L.)

**Keywords:** multi-manipulator systems, shared workspace environment, collision avoidance, multi-agent deep reinforcement learning, centralized training and decentralized execution, motion planning

## Abstract

In multi-manipulator systems operating within shared workspaces, achieving collision-free posture control is challenging due to high degrees of freedom and complex inter-manipulator interactions. Traditional motion planning methods often struggle with scalability and computational efficiency in such settings, motivating the need for learning-based approaches. This paper presents a multi-agent deep reinforcement learning (MADRL) framework for real-time collision-free posture control of multiple manipulators. The proposed method employs a line-segment representation of manipulator links to enable efficient interlink distance computation to guide cooperative collision avoidance. Employing a centralized training and decentralized execution (CTDE) framework, the approach leverages global state information during training, while enabling each manipulator to rely on local observations for real-time collision-free trajectory planning. By integrating efficient state representation with a scalable training paradigm, the proposed framework provides a principled foundation for addressing coordination challenges in dense industrial workspaces. The approach is implemented and validated in NVIDIA Isaac Sim across various overlapping workspace scenarios. Compared to conventional state representations, the proposed method achieves faster learning convergence and superior computational efficiency. In pick-and-place tasks, collaborative multi-manipulator control reduces task completion time by over 50% compared to single-manipulator operation, while maintaining high success rates (>83%) under dense workspace conditions. These results confirm the effectiveness and scalability of the proposed framework for real-time, collision-free multi-manipulator control.

## 1. Introduction

Multi-manipulator systems [1,2,3] provide significant benefits for complex collaborative tasks and simple repetitive operations, especially in applications like assembly [4] and cooperative transportation [5]. In environments where multiple manipulators operate within a shared workspace, collision-avoidance motion planning remains a critical challenge [6]. Traditional motion planning techniques are primarily categorized into two types: sampling-based and optimization-based approaches. Sampling-based methods [7], such as rapidly exploring random trees (RRT) [8] and the probabilistic roadmap method (PRM) [9], attempt to find collision-free trajectories directly within the manipulator’s configuration space (C-space) by connecting randomly sampled points to construct feasible paths. Although these techniques are generally effective for straightforward path-planning tasks, their uniform sampling across the entire space often results in high computational costs. Moreover, they frequently struggle in complex environments characterized by dynamic constraints or task-specific demands. To overcome these limitations, advanced variants like kino-dynamic RRT [10] and Bi-RRT [11] have been developed; however, these improvements led to increased algorithmic complexity and higher computational requirements.

In contrast, optimization-based methods [12], including CHOMP [13] and TrajOpt [14], offer the advantage of seamlessly integrating task specifications and system dynamics by formulating motion planning as a constrained optimization problem. Nevertheless, these methods typically pose path planning as a nonlinear and nonconvex optimization issue [15], resulting in elevated computational costs and decreased convergence stability. Specifically, the solutions heavily rely on the quality of the initial trajectory, often converging to local optima [16,17]. Consequently, most existing studies have employed an offline planning strategy that decouples motion planning from the sensing and actuation phases. Although these methodologies perform well in structured, static environments, they prove inadequate for dynamic situations necessitating real-time responsiveness [18].

Moving beyond traditional motion planning approaches, deep reinforcement learning (DRL) techniques have been widely adopted for robot path planning [19,20,21]. Cheng et al. [22] developed a DRL-based algorithm to train a manipulator to navigate to a target position while avoiding dynamic obstacles. Their research introduced a key point-based state representation for both the manipulator and the moving obstacle, alongside a reward function determined by the distances between predefined key points, thereby enabling real-time path planning for dynamic obstacle avoidance. Nonetheless, the single-manipulator setup limits scalability in multi-manipulator systems where inter-agent collision avoidance is critical. In addition, recent studies in reinforcement learning and neural adaptive control have expanded the applicability of learning-based approaches to broader control challenges. For instance, self-triggered approximate optimal neuro-control via adaptive dynamic programming has achieved efficient and stable control of nonlinear systems [23]. Adaptive critic design has also been applied to ensure safety-optimal fault-tolerant control under asymmetric input constraints in unknown nonlinear systems [24]. Moreover, noise suppression zeroing neural networks have been introduced to robustly solve time-varying inverse kinematics problems of mobile manipulators under external disturbances [25]. These works demonstrate the potential of integrating advanced learning-based control strategies into multi-agent motion planning frameworks, particularly for enhancing safety, robustness, and scalability in complex robotic environments.

By extending DRL beyond single-robot configurations, researchers have explored its implementation in motion planning for multirobot systems [26,27,28]. Multi-agent deep reinforcement learning (MADRL) [29,30] permits each agent to independently learn its policy while also modeling inter-agent cooperation and interactions, achieving effective performance in complex task environments. Indeed, the effectiveness of MADRL has been demonstrated in a variety of multi-agent systems. Recent studies have shown its success in coordinating UAV swarms [31,32] and quadrupedal robot teams [33], and dual-arm robotic assembly tasks [34], while Samak et al. [35] utilized MADRL in both cooperative and competitive contexts involving autonomous vehicles, successfully managing complex driving tasks such as navigating intersections and autonomous racing. In their study, each vehicle developed its policy based solely on partial observations, and the decentralized reinforcement learning framework demonstrated the feasibility of real-time learning and control. In addition, Wang et al. [36] proposed a MUTS-based cooperative target stalking framework for multi-USV systems, where multi-agent deep reinforcement learning is utilized to coordinate multiple unmanned surface vehicles for efficient target pursuit and collision avoidance. However, transferring such approaches from robot navigation to manipulator systems remains difficult owing to the manipulators’ higher degrees of freedom and more complex kinematic structures.

In this study, we introduce a MADRL-based solution for collision-free posture control of muti-manipulators operating within a shared workspace environment (SWE). The principal contributions of this study are as follows.

1. Line segment-based posture representation: A compact link representation is proposed by modeling each manipulator link as a line segment tailored to their cylindrical structures, facilitating a state representation optimized for collision detection and distance computation. This structure enables efficient and precise computation of interlink distances, allowing posture states to be optimized for collision avoidance. A distance-based reward is incorporated to promote early avoidance behavior, ensuring both computational efficiency and accurate collision-free posture control.
2. Application and validation of a centralized training and decentralized execution architecture for cooperative posture control: Inspired by its success in other robotic domains like UAV swarms and ground robots [31,32,33,34,35,36], we apply and validate a MADRL planner based on centralized training and decentralized execution (CTDE) framework for the unique challenges of multi-manipulator systems. CTDE is a widely adopted approach [37,38,39] in multi-agent reinforcement learning, enabling agents to leverage global information during training while executing decentralized policies based solely on local observations. In our approach, a centralized critic leverages global state information during training to learn coordinated strategies. Subsequently, during execution, each manipulator acts on its learned policy using only local observations. This work demonstrates the suitability of the CTDE framework for solving intricate collision avoidance and coordination problems in the constrained, shared workspace of multi-manipulators.

## 2. Materials and Methods

### 2.1. System Overview

**Figure 1:**
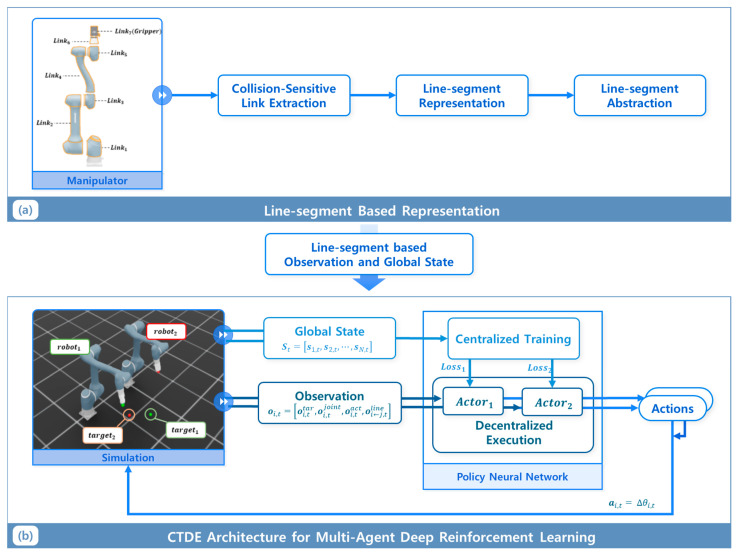
Overall architecture of the proposed method (**a**) line-segment representation and (**b**) multi-agent deep reinforcement learning based on CTDE.

In this section, we provide an overview of the proposed framework. In this paper, we present a MADRL framework for motion planning within a multi-manipulator system operating in SWEs. As illustrated in Figure 1, the comprehensive framework incorporates (a) a learning methodology that employs line segment-based representation for manipulators and (b) a MADRL planner based on CTDE architecture. A line segment-based model represents each manipulator link as a line segment, which is critical for effectively capturing the spatial arrangement of manipulators within shared workspaces while maintaining low computational cost. By computing the distances between these line segments, the system can assign distance-based penalties or rewards, thereby promoting the development of collision avoidance strategies among agents. The CTDE architecture enables each manipulator to learn cooperative motion policies through a centralized critic that leverages the global state—comprising the positions, velocities, and motion trajectories of all manipulators during training. By incorporating this comprehensive information, the critic can accurately evaluate joint interactions among agents, facilitating the actor’s learning of coordinated and collision-free behaviors. During execution, however, each manipulator operates in a decentralized manner, relying solely on its local observations to make decisions. This configuration is particularly advantageous for multi-manipulator systems, where manipulators must not only identify each other’s positions during training but also autonomously detect and avoid collisions during operation. The entire system is implemented within a simulation environment using NVIDIA Isaac Sim 4.5, and reinforcement learning is performed using Isaac Lab. The framework is validated through extensive training and evaluation across diverse scenarios.

### 2.2. Line Segment-Based Representation of Manipulator Links

This section introduces a state representation specifically designed for inter-manipulator collision avoidance in SWEs. The proposed method models each manipulator link as a line segment, facilitating the identification of spatial relationships among manipulators. This representation is integrated into the reinforcement learning state, enabling agents to effectively develop collision-avoidance strategies. A serial manipulator typically comprises n+1 links and n joints, where *n* denotes the number of links, often corresponding to the manipulator’s degrees of freedom. Excluding the base link (${Link}_{0}$), the set of links is defined as follows:

(1) $$Link=\{{Link}_{1},\;{Link}_{2},\cdots ,\;{Link}_{n}\}$$

Among these links, those rigidly attached to the base and contributing minimally to collision-sensitive motion have a negligible likelihood of entering other manipulators’ workspaces. Consequently, such links are excluded when defining collision-sensitive regions. The parameter m is introduced to specify the starting index of the links considered for collision detection. If the manipulator is equipped with a gripper, it is explicitly included in the collision-sensitive set because its shape and motion directly affect spatial overlap with neighboring manipulators. Accordingly, the collision-sensitive link set is defined as

(2) $${Link}_{collision}=\left\{{Link}_{m},\;{Link}_{m+1},\cdots ,\;{Link}_{n},\;{Link}_{n+1}\right\},\;$$

where ${Link}_{m},\;{Link}_{m+1},\cdots ,\;{Link}_{n}$ denotes the set of manipulator body links that can contribute to potential collisions, and ${Link}_{n+1}$ represents the gripper. Each link is represented by a single line segment extracted along its principal axis, capturing the dominant geometric shape of the link. For link i, the endpoints defining the physical boundary along the principal axis are denoted as $p_{i}^{s}$ and $p_{i}^{e}$. The line segment representation of link i is defined as

(3) $$L_{i}=\left(p_{i}^{s},{\;p}_{i}^{e}\right),\;\left(i=m,m+1,\cdots ,n,\;n+1\right),$$

This concise representation effectively characterizes the spatial extent of each link while reducing computational complexity. To further simplify the representation, links sharing the same joint axis are combined into a single segment. By representing dynamically coupled links as single-segment sets, the state representation becomes more compact while maintaining the essential geometric and kinematic properties necessary for collision avoidance.

**Figure 2:**
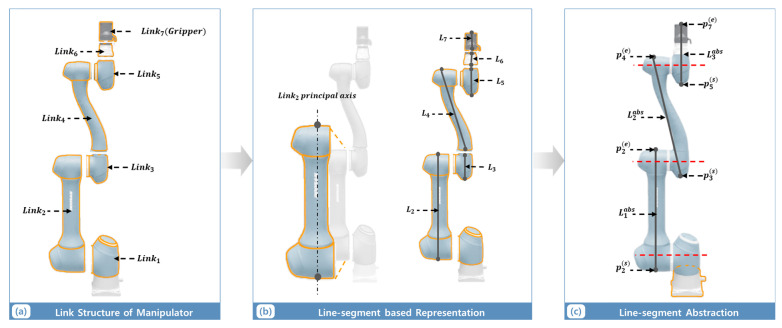
Abstraction Pipeline for Line-Segment Based Manipulator Representation. (**a**) link structure of manipulator, (**b**) line-segment based representation, (**c**) line-segment abstraction. The red dashed line represents the axis of the manipulator joint.

In this study, we employ a six-degree-of-freedom (6-DoF) manipulator with a gripper, as shown in Figure 2. ${Link}_{1}$ is rigidly attached to the base and contributes minimally to collision-sensitive motion; hence, it is excluded from set of ${Link}_{collision}$. The collision-sensitive region extends from ${Link}_{2}$ through ${Link}_{6}$ and the gripper. Each link is depicted by a single line segment aligned with its principal axis, defined by the two endpoints of its physical boundary. ${Link}_{2}$ undergoes independent rotational motion about its joint and is thus represented by a single line segment reflecting its individual kinematic behavior. In contrast, ${Link}_{3}$ and ${Link}_{4}$ share a common joint axis and exhibit coupled motion; consequently, they are combined and represented by a single line segment that captures their integrated movement. Similarly, ${Link}_{5}$ and ${Link}_{6}$, along with the gripper, serve as integrated end effectors and are modeled as representative line segments. Consequently, out of the six links comprising the manipulator, three representative line segments are selected to represent ${Link}_{2}$ through ${Link}_{6}$, including the gripper region. These chosen line segments effectively capture the areas within the defined workspace that are most susceptible to inter-manipulator collisions, as illustrated in Figure 2.

This representation enables efficient distance computation between manipulator links and serves as a crucial component in multi-agent reinforcement learning for collision avoidance.

### 2.3. Observation and Global State

Building upon the line segment-based representation introduced in Section 2.2, we define the observation and global state structures used in our reinforcement learning framework. In this study, an observation refers to the local information directly perceivable by each agent, while the global state represents the integrated state information of all agents. This formulation adheres to the CTDE architecture, wherein a centralized value function is trained using the global state during learning, and each agent independently executes its policy based solely on its own observations during runtime. This architecture enables agents to develop cooperative strategies and collision avoidance behaviors during training by leveraging shared information while maintaining fully decentralized control during execution.

#### 2.3.1. Observation Space

In this subsection, we first describe the structure of the observation space, which defines the information each agent perceives from itself and the environment. The observation of each manipulator comprises data about the agent and its surrounding environment, which the policy utilizes for collision avoidance. The vector between the manipulator gripper and targets $o_{i,t}^{tar}$ is defined in Equation (4):

(4) $$o_{i,t}^{tar}=(p_{i}^{tar}-p_{i,t}^{grip}),$$

where $p_{i}^{tar}$, denotes the target position of manipulator i, which remains fixed during each episode, and $p_{i,t}^{grip}$ denotes the position of end point of manipulator’s gripper at time t.

The joint state for manipulator i, $o_{i,t}^{joint}$, is defined using Equation (5):

(5) $$o_{i,t}^{joint}\;=\;{[\theta }_{i,t},\;{\dot{\theta }}_{i,t}],$$

where $\theta_{i,t},\;$ and ${\dot{\theta }}_{i,t}$, represent the joint positions and the joint velocities of its n degrees of freedom of manipulator, respectively. This component captures the manipulator’s current state of motion and provides essential information for stable control. The previous action, $o_{i,t}^{act}$, is defined by Equation (6):

(6) $$o_{i,t}^{act}\;=\;a_{i,t-1}\in R^{6},$$

which corresponds to the action vector executed by manipulator i at time step t−1. This is included to maintain temporal consistency in the policy and reflect inertia in the agent’s behavior. The relative link segment information from manipulator j to manipulator i, $o_{i\leftarrow j,t}^{line}$, is defined in Equation (7):

(7) $$o_{i\leftarrow j,t}^{line}\;=\;L_{i\leftarrow j,k,t}^{abs},\;(k=1,2,3)$$

where j denotes the index of the other manipulators. Each element denotes the direction vector of one of the three link segments of manipulator j (indexed by k = 1, 2, 3), transformed into the local coordinate frame of manipulator i. Since the policy operates at the individual agent level without access to global information during execution, providing observations in a consistent, agent-centric manner is crucial. By converting the relative link segment data of other manipulators into the local coordinate frame of each agent, we maintain an invariant input structure regardless of the absolute position of the agent or orientation in the global space. This representation facilitates stable decentralized policy learning and enhances the generalization capabilities of the policy across diverse spatial configurations. In summary, the complete observation perceived by manipulator i at time step t comprises the following elements:

(8) $$o_{i,t}=\left[o_{i,t}^{tar},o_{i,t}^{joint},o_{i,t}^{act},o_{i\leftarrow j,t}^{line}\right]$$

#### 2.3.2. Global State Space

In this section, we explain the structure and role of the global state space within the proposed framework. The global state aggregates the state information of all agents and serves as input to the centralized value function, enabling value estimation that comprehensively accounts for the overall environment. This allows for the evaluation of how individual agent actions impact the entire system, thereby enhancing training stability and promoting collision avoidance behaviors among agents. The training process utilizes the full state information of all agents, and the global state $S_{t}$ at time step t is defined as follows:

(9) $$S_{t}=\left[s_{1,t},s_{2,t},\;\cdots ,s_{N,t}\right],\;(i=1,2,\cdots ,N),$$

where *N* denotes the number of agents, and $s_{i,t}$ represents the state vectors of agent i at time step t. The structure of $s_{i,t}$ is defined in Equation (10):

(10) $$s_{i,\;t}=\left[o_{i,t}^{tar},o_{i,t}^{joint},o_{i,t}^{act},\;s_{i,t}^{line}\;\right],$$

where $o_{i,t}^{tar},o_{i,t}^{joint}$, and $o_{i,t}^{act}$ reuse the components defined in Section 2.3.1 for local observations. The link segment information of manipulators i, $s_{i,t}^{line},$ is defined as Equation (11):

(11) $$s_{i,t}^{line}\;=\;L_{i,k,t}^{abs},\;(k=1,2,3)$$

In contrast to the observation space—where the relative positions of neighboring manipulators are transformed into the local coordinate frame of each agent—the manipulator position information within the global state is represented in the global coordinate frame. Using a global coordinate frame enables a centralized value function to evaluate the positions and orientations of all agents within a single reference system. This allows a thorough assessment of the manipulators’ absolute spatial configurations, thereby enhancing the accuracy of value estimation. As a result, the learning process more effectively captures inter-agent dependencies and improves collision-avoidance behaviors. This global state formulation underpins centralized value estimation and is essential for promoting cooperative behaviors and ensuring safety in multi-agent SWEs.

### 2.4. Action Space

In this subsection, we describe the action space that defines how each agent interacts with the environment. In this study, a 6-DOF manipulator is utilized, with each agent’s action defined as the joint position control of the manipulator. At each time step, the policy network (actor) generates a six-dimensional continuous action vector $a_{t}$, representing the relative displacement of joint positions. This action vector $a_{t}$ is interpreted as the joint position increment $∆\theta_{t}$, defined as follows:

(12) $$a_{t}={∆\theta }_{t}\in R^{6}$$

This increment is directly added to the current joint position $\theta_{t}$ without additional scaling. To prevent exceeding physical joint limits, a clipping operation is applied within the lower and upper joint bounds when computing the next joint position $\theta_{t+1}$, which is then sent as a joint position command to the manipulator system. The complete process can be defined as follows:

(13) $$\theta_{t+1}=clip\left(\theta_{t}+{∆\theta }_{t},\theta_{min},\theta_{max}\right)$$

### 2.5. Reward Function Design

In this section, we present the design of the reward function, which plays a critical role in guiding the learning process of each agent. The reward function is designed to encourage the manipulator to reach its target efficiently while avoiding collisions with other manipulators in the SWE. The overall reward consists of five components: distance-to-target, action penalty, target-reaching, avoidance, and collision penalties.

*Distance-to-Target reward* ($R_{dist,t}$): This reward is calculated as the inverse of the squared Euclidean distance between the position of end point of manipulator’s gripper $p_{grip,t}$ and the target position ($p_{tar}$). It incentivizes the manipulator to approach the target by awarding higher rewards as the end effector nears the target.

(14) $${\;R}_{dist,t}={\left(\frac{1}{1+{\left\|p_{grip,t}-p_{tar}\right\|}^{2}}\right)}^{2}$$

*Action Penalty* ($R_{action\_penalty}$): This penalty promotes temporal smoothness in actions, discouraging abrupt changes that could lead to unstable behavior. Here, $a_{t}$ denotes the 6-dimensional joint-space action vector of the manipulator at time step t.

(15) $$R_{action\_penalty}={\left\|a_{t}-a_{t-1}\right\|}^{2}$$

*Target-Reaching Reward* ($R_{target}$): An additional reward is granted when the distance between the end point of manipulator’s gripper and the target falls below a predefined threshold ($\delta_{target}$). This mechanism ensures the manipulator does not focus solely on collision avoidance at the expense of achieving the target, thereby promoting both task success and safety.

(16) $$R_{target}=\left\{\begin{array}{c} \;1.0,\;\mathrm{if}\;{\left\|p_{grip,t}-p_{tar}\right\|}^{2}\leq \delta_{target}\\ 0.0,\;otherwise\; \end{array}\right.$$

In our experiments, the threshold is set to $\delta_{target}=0.05\;m$.

*Avoidance Reward* ($R_{avoid,t}$): This reward assigns a continuous negative value based on the proximity between the link segments of different manipulators. As the distance $d_{ij}^{\left(m,n\right)}$ between link m of manipulator i and link n of manipulator j approaches the collision threshold $\delta_{collision}$, the penalty increases. Here, m, n correspond to the three line-segments defined for each manipulator, as illustrated in Figure 2. This design encourages early avoidance behavior, and not just immediate collision prevention.

(17) $$R_{avoid,t}=-\sum_{i\neq j}\sum_{m=1}^{3}\sum_{n=1}^{3}max\left(0,\;\frac{\delta_{collision}-d_{ij}^{\left(m,n\right)}}{\delta_{collision}}\right)$$

*Collision Penalty* ($R_{collision}$): This penalty is applied when the minimum distance between any two link segments $d_{ij}^{\left(m,n\right)}$ falls below the collision threshold $\delta_{collision}$, explicitly discouraging collisions between manipulators.

(18) $$R_{collision}=\left\{\begin{array}{cc} -2.0, & \;\mathrm{if}\;d_{ij}^{\left(m,n\right)}\leq \delta_{collision}\\ 0.0, & \;otherwise \end{array}\right.\;for\;all\;i\neq j,\;m,\;n\;\in \;\{1,2,3\}$$

The collision threshold $\delta_{collision}$ is defined as:

(19) $$\delta_{collision}\;=\;D\;+\;\varepsilon ,$$

where D denotes the diameter of the cylindrical link, and ε represents a safety margin to ensure sufficient clearance beyond physical contact. In our experiments, we set ε=0.05 m. We analyze the effect of different values of ε. When the margin is too small, collisions occur frequently, reducing the overall success rate. In contrast, when the margin is too large, the agents adapt overly conservative strategies, often failing to reach their goals within the time limit. Based on this trade-off, we select a moderate margin value that provides the best balance between collision avoidance and task success and apply this setting in our experiments.

This reward structure enables each manipulator to not only reach its target but also maintain safe distances from others by perceiving proximity in real time. The total reward consists of a weighted sum of components, along with conditionally applied penalties and bonuses, defined as follows:

(20) $$R=\omega_{dist,t}\cdot R_{dist,t}+\omega_{action\_penalty}\cdot R_{action\_penalty}+{\omega_{avoid,t}\cdot R}_{avoid,t}+\;R_{collision}+R_{target}$$

In our experiments, the threshold is set to $\omega_{dist,t}=1.0$, $\omega_{action\_penalty}=-0.0001$, and $\omega_{avoid,t}=5.0$. To determine the coefficients, we conduct iterative experiments under different environmental conditions. We observe that increasing the distance reward weight strengthens goal-reaching behavior but can lead to higher collision risk. On the other hand, larger avoidance reward values reduce collisions but make the agent overly conservative, which occasionally prevents timely goal arrival. A strong action penalty restricts maneuverability in dense environments, while a weaker penalty provides a better balance between smoothness and agility. The weights are adjusted to balance obstacle avoidance and goal-reaching performance. The chosen coefficients are determined based on sensitivity analyses, as detailed in Section 3.1.3.

### 2.6. MAPPO Learning Approach

**Figure 3:**
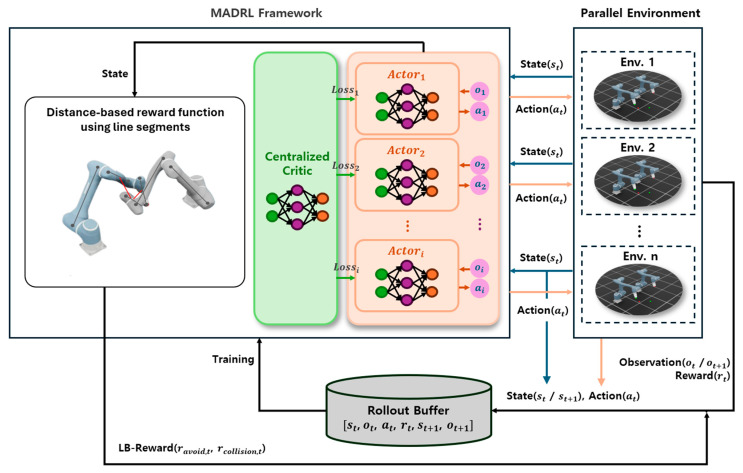
The proposed MADRL framework. The Parallel Environment module illustrates the parallelized training setup, in which n independent environments, each involving i cooperative agents, simultaneously generate diverse experiences. The MADRL Framework module depicts the policy learning process within a single environment composed of i agents, consisting of a centralized critic and multiple decentralized actors. Collected trajectories from all environments are stored in a shared rollout buffer and subsequently used for policy updates.

In this subsection, we describe the learning algorithm adopted in our framework and explain how it addresses the challenges of multi-agent settings. This study employs multi-agent deep reinforcement learning (MADRL) framework as shown in Figure 3. In MADRL, a critical challenge is the non-stationarity of the environment, which arises as each agent’s policy is updated concurrently, thereby violating the Markov assumption [40]. The CTDE architecture adopted in this study is one of the effective methodologies for addressing this non-stationarity problem in multi-agent systems (MAS). Within the CTDE paradigm, the centralized critic has access to global information—the observations and actions of all agents—during the training phase. This allows the critic to maintain a stationary learning perspective, as it learns from complete information that accounts for all policy changes. By providing a stable learning signal to each decentralized actor, this centralized critic effectively mitigates the non-stationarity problem that each agent would otherwise face individually. While other techniques exist to improve stability for independent learners, such as enhancing experience replay [41,42], we choose the CTDE paradigm as it offers a more fundamental, architectural solution to the non-stationarity problem.

To realize this approach, we utilize the multi-agent proximal policy optimization (MAPPO) algorithm [43], an extension of the original proximal policy optimization (PPO) algorithm [44] tailored for multi-agent environments. MAPPO leverages PPO’s clipping-based policy update mechanism to enhance training stability in multi-agent settings. MAPPO comprises two distinct neural networks: a policy network (the actor) parameterized by θ, and a value function network (the critic) with parameters φ. The overall architecture of the proposed MADRL framework is illustrated in Figure 3.

Both the policy and value networks are implemented as multilayer perceptrons (MLPs) with four hidden layers containing 512, 512, 256, and 128 units. To introduce nonlinearity and improve training stability, the exponential linear unit activation function is applied to all layers. The policy network receives each agent’s local observation $o_{i}$ as input and outputs the parameters of the action distribution. Assuming a continuous action space, the network generates the mean and log standard deviations of a multivariate Gaussian distribution, from which the actual action $a_{i}$ is sampled. In contrast, the value function network (critic) leverages the global state S by integrating all agents’ states as inputs, outputting a scalar that estimates the return from that state. This value is used to evaluate the current policy and compute the advantages for policy updates.

Although the policy network relies solely on each agent’s local observations, the value network utilizes the full global state. This architecture enables accurate value estimation during training, where global information is accessible, and allows decentralized execution during testing, with each agent acting independently based on its own observations. This CTDE framework boosts learning efficiency and execution flexibility in multi-agent environments. During training, interactions occur within a parallel simulation environment. At each time step, the global state $s_{t}$ is provided, and decentralized policy networks select actions $a_{i,t}$ based on local observations $o_{i,t}$. Rewards are then computed using Equation (21), and the environment transitions to the next global state $s_{t+1}$ based on the chosen actions, establishing an iterative interaction loop.

The transition data ${[s}_{t},o_{t},a_{t},r_{t},\;s_{t+1},o_{t+1}]$ are stored in a rollout buffer. Unlike off-policy algorithms that maintain large replay memories, MAPPO [42] employs an on-policy buffer that collects trajectories over a fixed time horizon before each update. Once the buffer reaches the predefined rollout length T, advantages are estimated using the generalized advantage estimation method to reduce variance while preserving low bias, and gradients for both the policy and value networks are computed. Their parameters are subsequently updated using the adam optimizer. The policy network ($\pi_{\theta }$) is optimized to maximize the following objective function:

(21) $$L\left(\theta \right)=\;[\frac{1}{Bn}\sum_{i=1}^{B}\sum_{k=1}^{n}min(r_{\theta ,i}^{\left(k\right)}A_{i}^{\left(k\right)},clip(r_{\theta ,i}^{\left(k\right)},\;1-\varepsilon ,\;1+\varepsilon )A_{i}^{\left(k\right)})]\;+\;\sigma \frac{1}{Bn}\sum_{i=1}^{B}\sum_{k=1}^{n}S[\pi_{\theta }(o_{i}^{\left(k\right)})]\;,$$

where $A_{i}^{\left(k\right)}$ denotes the advantage estimated via the Generalized Advantage Estimation (GAE) method, S represents policy entropy, and σ is the entropy coefficient hyperparameter. The parameter $r_{\theta ,i}^{\left(k\right)}$ signifies the importance sampling ratio, defined as

(22) $$r_{\theta ,i}^{\left(k\right)}=\frac{\pi_{\theta }(a_{i}^{\left(k\right)}\left|o_{i}^{k}\right.)}{\pi_{\theta_{old}}(a_{i}^{\left(k\right)}\left|o_{i}^{k}\right.)},$$

where $\theta_{old}$ refers to the parameters of the preceding policy. The value function network ($V_{\varphi }$) is trained to minimize the subsequent loss function:

(23) $$L\left(\varphi \right)=\;\frac{1}{Bn}\sum_{i=1}^{B}\sum_{k=1}^{n}(max[{\left(V_{\varphi }(s_{i}^{\left(k\right)})-{\hat{R}}_{i}\right)}^{2},(clip(V_{\varphi }(s_{i}^{\left(k\right)}),V_{\varphi_{old}}(s_{i}^{\left(k\right)})-\varepsilon ,\;V_{\varphi_{old}}(s_{i}^{\left(k\right)})+\varepsilon )-{\hat{R}}_{i}{)}^{2}]),$$

where ${\hat{R}}_{i}$ is the discounted reward in both loss functions, *B* refers to the batch size and *n* is the number of agents.

Training is conducted using the MAPPO algorithm [43], with key parameters outlined in Table 1. The training process spans 36,000 steps and utilizes 1024 parallel environment instances to enhance training efficiency. These settings are chosen to balance hardware constraints with training efficiency, ensuring stable convergence within a reasonable wall-clock time. In our implementation, the rollout length is explicitly set to a fixed number of steps per environment. With multiple environments running in parallel, the total number of transitions accumulated in the buffer before each update is determined by the product of the rollout length and the number of parallel environments. The collected batch is then divided into several mini-batches and reused for multiple learning epochs, enabling efficient utilization of the sampled experience. This effective buffer size ensures sufficient trajectory diversity across agents and environments, thereby improving training stability.

## 3. Results

### 3.1. Experimental Setup

**Figure 4:**
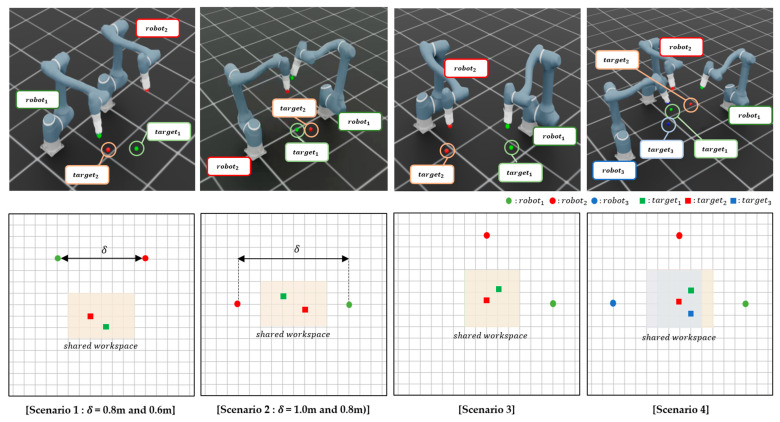
Four simulation environments used for reinforcement learning.

In this section, we describe the experimental setup used to evaluate the proposed method. In this study, a simulation environment is constructed using NVIDIA Isaac Sim. The Doosan Robotics A0912 model (Doosan Robotics, Suwon, Republic of Korea) is used as a manipulator, featuring six degrees of freedom and a working radius of 1200 mm. This manipulator is integrated into the Isaac Sim environment in accordance with its actual hardware specifications. DRL is conducted using Isaac Lab. Simulations are performed on a system equipped with an NVIDIA GeForce RTX 4070 Ti GPU (Nvidia, Santa Clara, CA, USA) and an Intel Core i7-14700K CPU (Intel, Santa Clara, CA, USA). Four representative scenarios with varying difficulty levels are designed to evaluate the proposed method within diverse SWEs, as illustrated in Figure 4.

- *Scenario 1 (Parallel Forward Configuration):* Two manipulators are placed side by side, both oriented in the same forward direction, and required to navigate toward their respective targets located ahead. The inter-manipulator distance is reduced to increase difficulty, thereby increasing the risk of collision.
- *Scenario 2 (Face-to-Face Configuration):* The two manipulators are positioned facing each other, with their targets situated behind the opposing agent. This arrangement creates a head-on trajectory conflict and, similar to Scenario 1, the inter-manipulator distance is decreased to increase interaction intensity and planning complexity.
- *Scenario 3 (Crossed Configuration, Two Manipulators):* Two manipulators are arranged diagonally so that their target trajectories intersect at the center of the workspace. This scenario introduces dynamic path-crossing behavior, necessitating precise collision avoidance within a constrained shared space.
- *Scenario 4 (Crossed Configuration, Three Manipulators):* Expanding upon Scenario 3, this setup incorporates an additional manipulator into the crossed arrangement, resulting in three manipulators concurrently navigating intersecting trajectories. This creates a highly complex and congested shared workspace, presenting a challenging scenario that tests the scalability and robustness of the proposed method in multi-manipulator coordination.

These four scenarios are meticulously crafted to replicate various realistic shared workspace configurations, enabling a systematic evaluation of the proposed multi-agent reinforcement learning framework under diverse spatial constraints and interaction complexities.

#### 3.1.1. Reinforcement Learning Environment Setup

In this subsection, we describe the reinforcement learning environment, including the task objectives, manipulator placement, and episode configuration. The reinforcement learning objective for each manipulator is to reach its target position without colliding with others. In all scenarios, the simulation environment includes manipulators and their respective target positions. Each manipulator is positioned at a designated grid point within the shared workspace, organized in a grid-based layout as shown in Figure 4. The relative spacing between manipulators is parameterized by *δ*, representing the inter-manipulator distance defined by the grid configuration. At the beginning of each episode, the initial joint angles of each manipulator are randomly sampled within predefined ranges to ensure a collision-free configuration. Initial joint velocities are set to 0 rad/s, maintaining a stationary initial state. The target position for each manipulator is randomly selected from the designated shared workspace, which is also organized in a grid-based layout as depicted in Figure 4. To further promote diversity and realism, the cube positions are sampled from bounded regions within the shared workspace, with the exact ranges varying across scenarios. Likewise, the base joint rotations are constrained to scenario-specific feasible intervals to avoid inter-manipulator collisions, while the second joint of every robot is restricted to [−0.43, 0.43] rad to prevent ground contact. All other joints are initialized randomly within their respective physical limits. This sampling is performed independently in each episode to promote task diversity and prevent overfitting to fixed trajectories. Each episode terminates under one of three conditions: all manipulators successfully reach their designated target positions within a defined tolerance threshold, the number of simulation steps exceeds a predetermined maximum limit, or any pair of manipulators collide within the shared workspace. Unlike traditional multi-agent training frameworks, which typically reset the entire environment after a single failure, this study employs a partial reset mechanism. Specifically, when a manipulator collides with the ground, only that manipulator is reinitialized to a new initial state, allowing the remaining manipulators to continue the current episode uninterrupted. This strategy aims to enhance training efficiency by minimizing disruptions to the learning process of unaffected agents caused by localized failures.

#### 3.1.2. Evaluation Metrics

The evaluation metrics for quantitative performance assessment are summarized in Table 2.

#### 3.1.3. Sensitivity Analysis

To validate the robustness and appropriateness of the proposed reward design and safety parameters, we conduct a series of sensitivity analyses focusing on two key aspects: the safety margin parameter (ϵ) and the reward weight coefficients ($\omega_{i}$). These experiments aim to quantitatively evaluate how different parameter choices influence task performance, including success rate and collision avoidance capability. We first investigate the effect of varying the safety margin ϵ, which defines the minimum allowable inter-robot distance during task execution. We test five values of ϵ = {0.01, 0.03, 0.05, 0.08, 0.10} while keeping all other conditions constant, as shown in Table 3. Each experiment is repeated for 100 independent trials to ensure statistical reliability. The results show that when the safety margin is too small (ϵ = 0.01), frequent collisions significantly reduce the success rate. Conversely, excessively large margins (ϵ ≥ 0.08) result in overly conservative behavior, causing agents to fail to reach the goal within the time limit. The optimal trade-off is observed at ϵ = 0.05, which consistently yields the highest success rate across all scenarios. This value is therefore adopted in subsequent experiments.

To further assess the robustness of the proposed reward design, we analyze how different reward weight configurations influence task performance. The coefficients (ω) are iteratively tuned to balance objectives under various environmental conditions, as shown in Table 4.

The results show that as the distance between robots decreases, success rates decline due to increased collision risk, highlighting the importance of precise reward tuning in dense workspaces. A larger distance reward improves goal-reaching performance in spacious settings but raises collision risk in confined ones, which must be counteracted by an appropriately weighted avoidance reward. While increasing the avoidance reward reduces collisions, excessive weighting leads to conservative behavior and task delays. Similarly, a strong action penalty enhances control stability but limits maneuverability, whereas a weaker penalty provides a better balance. Based on these insights, we adapt the following configuration for final training: a distance reward weight of 1.0, an action penalty of −0.0001, and an avoidance reward of 5.0, which together achieve a balance between goal-reaching, safety, and agility.

### 3.2. Performance of the Proposed Method Under Various Environmental Conditions

**Figure 5:**
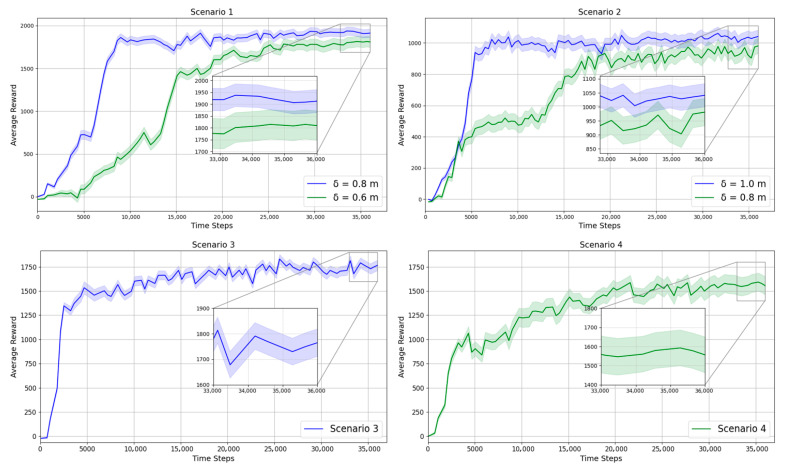
Average reward value of each round step across all scenarios.

To evaluate the robustness of the proposed method across diverse environmental settings, experiments are conducted in the four scenarios illustrated in Figure 4. Table 5 and Figure 5 display the performance of the proposed method across these scenarios.

The learning curves in Figure 5 show the mean reward from 100 independent runs with different random seeds. For each run, the random seed is applied to both the initial cube placements and the joint postures of the manipulators, sampled within their physically feasible ranges, to ensure diversity and avoid bias toward specific scenarios. The shaded area in the shared workspace of Figure 4 represents the range of random cube placements.

**Figure 6:**
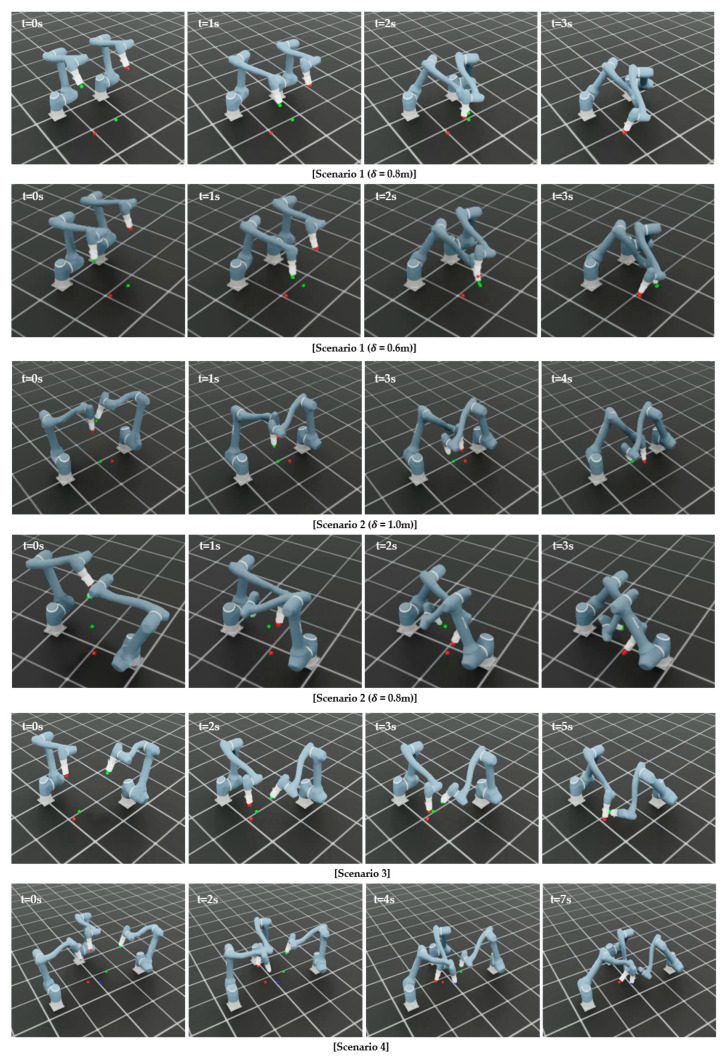
Visualization of successful cases across all scenarios.

The success rate generally declines with increasing environmental complexity (Table 5). When the inter-manipulator distance is reduced, the SR drops by 3–4%, and adding a third manipulator causes an additional 15% decrease. Failure analysis indicates that most failures result from collisions rather than timeouts, accounting for 4–7% and 2–4%, respectively, in Scenarios 1 and 2. Scenario 3 shows the highest stability with only 2% collisions and 1% timeouts, while Scenario 4 exhibits the greatest vulnerability with 12% collisions and 5% timeouts. The average reward follows a similar trend, decreasing by about 10% as task density increases. In terms of convergence steps (CS), environments with wider spacing converge within 8 k–15 k steps, whereas denser configurations require 19 k–27 k steps. Figure 6 show that the proposed method achieves stable convergence across all scenarios.

**Figure 7:**
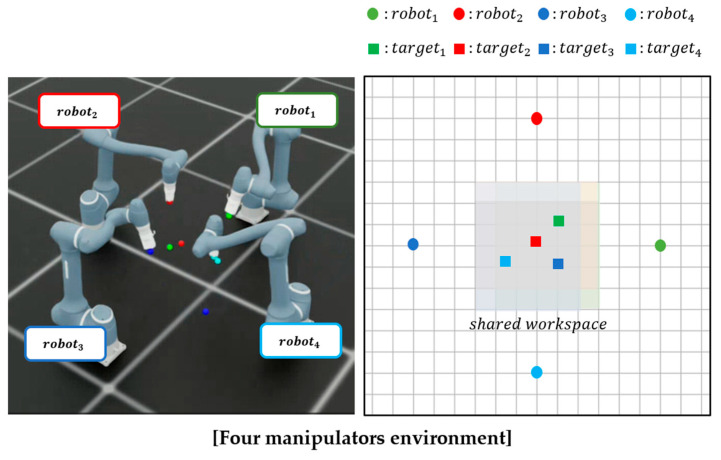
Four-manipulator simulation environments used for reinforcement learning.

**Figure 8:**
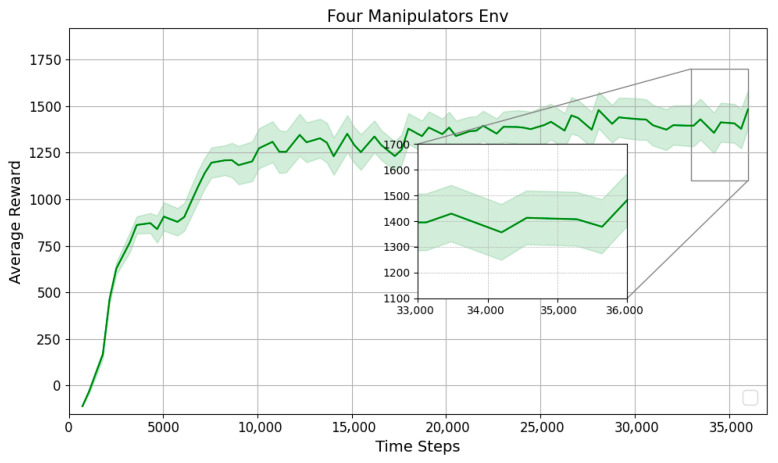
Average reward value of each round step across four manipulators environment.

To further assess the performance of the proposed method under more challenging conditions, additional tests are conducted with four manipulators as shown in Figure 7. Similar to the previous scenarios, 100 independent runs with different random seeds are performed to ensure diversity and avoid bias toward specific scenarios. Table 6 presents the performance of the proposed method in the four-manipulator environment. The learning curves in Figure 8 show the mean reward across these runs.

**Figure 9:**
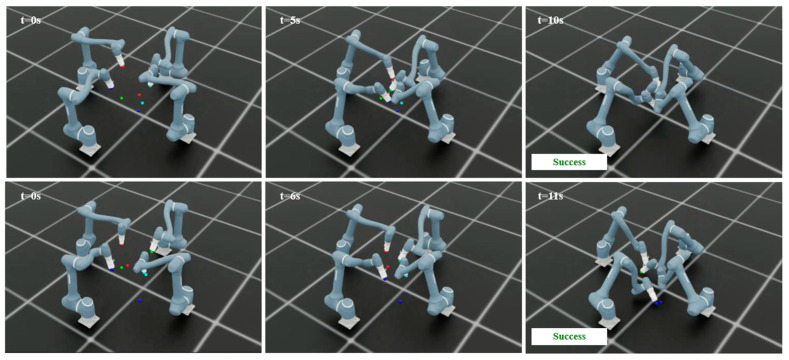
Visualizations of our method in four-manipulator scenarios (success cases).

In the four-manipulator scenario, the proposed method achieves an 80% success rate, indicating that most trials are completed successfully. Failures are mainly due to collisions (16%) and timeouts (4%), reflecting the increased coordination difficulty in dense environments. The average reward of 1485.38 (±15.79) confirms stable learning performance, while convergence is reached at approximately 23,000 steps. These results show that the algorithm effectively adapts to the increased complexity of multi-manipulator interactions. Figure 9 illustrates that the proposed method achieves successful training across all scenarios.

### 3.3. Performance Comparison with Reinforcement Learning Setups

#### 3.3.1. Performance Comparison with Independent Value Function

This section investigates the effect of value function ($V_{\varphi }$) design on learning performance in multi-agent environments. All experiments are performed under identical environmental conditions and state representations to ensure a fair comparison. In particular, the line segment-based (LS) representation is fixed as the baseline state encoding, so that the observed differences can be attributed solely to the design of the value function. The main difference lies in the structure of the value function ($V_{\varphi }$) used during training. The proposed method employs a centralized value function ($V_{\varphi }$) that incorporates the global state and actions of all agents during training. This centralized framework allows each agent to consider the behaviors of others, promoting the development of more coordinated and cooperative policies. In contrast, an alternative method estimates the value function ($V_{\varphi }$) solely based on local observations, resulting in independent learning among agents. The quantitative results from 100 trials for each scenario are summarized in Table 7. The independent learning setup is implemented using Independent Proximal Policy Optimization (IPPO) [45], while the centralized training approach utilizes MAPPO [43].

**Figure 10:**
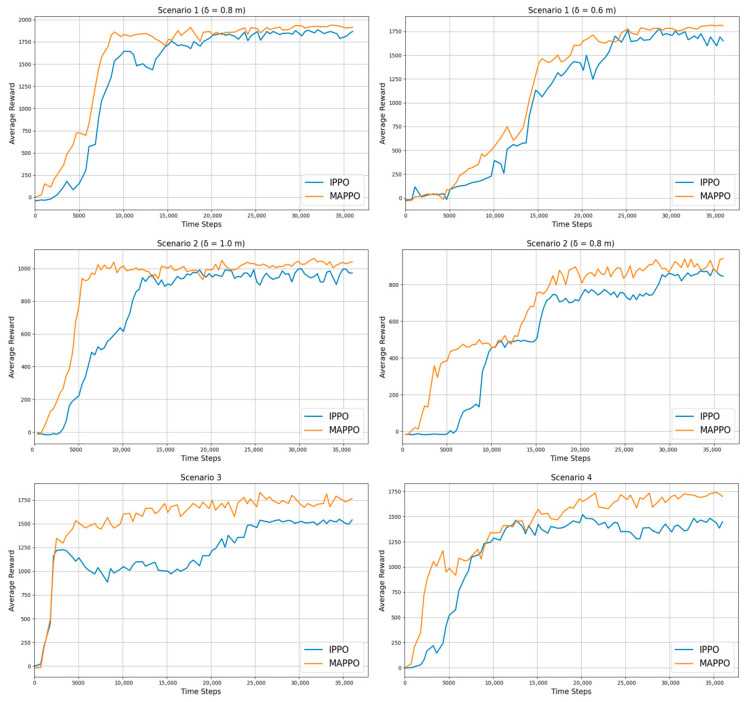
Average reward per step across all scenarios: IPPO vs. MAPPO.

Across all scenarios, the centralized learning approach consistently outperforms the independent one in both success rate and learning efficiency. In Scenarios 1 and 2, the centralized method improves success rates by 5–15 percentage points as the inter-manipulator distance decreases. In Scenarios 3 and 4, which involve two and three agents, the gains reach up to 17%. Moreover, the centralized critic achieves faster convergence, reducing computation steps by nearly half (e.g., from 19,940 to 10,800 steps in Scenario 1) while maintaining higher stability. Figure 10 illustrates that the centralized method converges more rapidly and exhibits smoother learning curves with lower variance compared to the independent learning approach.

#### 3.3.2. Performance Comparison with CTDE Algorithms

This section comparatively analyzes the performance of the proposed multi-manipulator framework against several representative CTDE algorithms, including HAPPO [46], HATRPO [46], and HAA2C [47], alongside the MAPPO [43] baseline. All methods are trained under identical experimental conditions and hyperparameters. Table 8 presents the quantitative comparison of policies across various environments.

**Figure 11:**
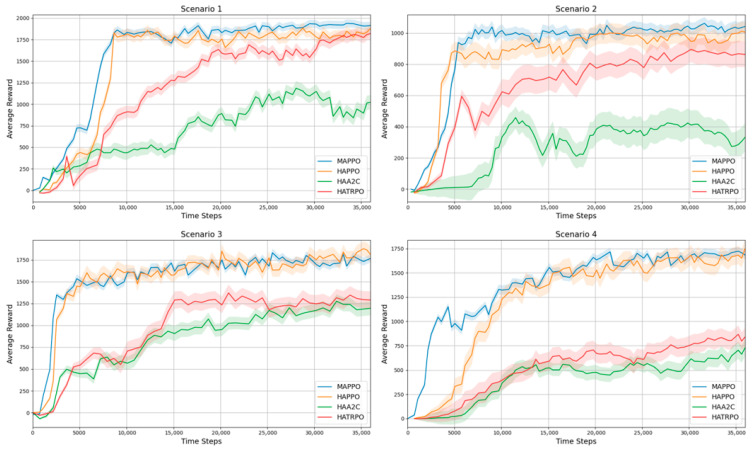
Comparisons of average reward of CTDE algorithms.

In Scenario 1 (δ = 0.8 m), all algorithms achieve over 90% success except HAA2C (84%). In Scenario 2 (δ = 1.0 m), MAPPO achieves the highest success rate (95%), while HAA2C shows the lowest (83%). In Scenario 3, MAPPO and HAPPO reach 97%, whereas HAA2C remains lower at 83%. Finally, in Scenario 4, success rates decrease across all methods, ranging from 76% (HAA2C) to 83% (MAPPO). Overall, MAPPO consistently achieves the highest success rate, followed by HAPPO, while HATRPO remains stable and HAA2C lags behind. Under identical experimental conditions, MAPPO demonstrates the fastest convergence speed and the highest average return. HAPPO shows comparable performance, whereas HATRPO maintains stable convergence with slightly lower returns. In contrast, HAA2C converges more slowly and achieves the lowest final returns among all algorithms. Figure 11 presents the average return curves across four representative scenarios.

#### 3.3.3. Performance Comparison with a Conventional Manipulator State Representation Method

**Figure 12:**
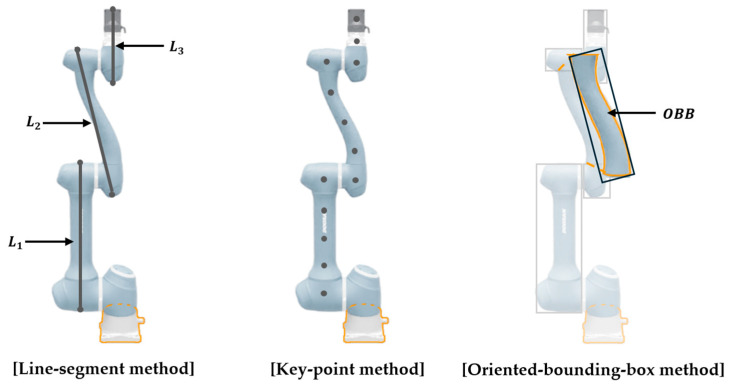
State representation of manipulator: line-segment (ours) vs. key point and oriented bounding box.

This section comparatively analyzes the proposed line-segment (LS) based state representation method with the traditional key-point (KP) based method introduced by Cheng [22], as well as an oriented bounding box (OBB) based state representation. These methods differ in how they represent the states of manipulator links. The LS approach models each link as a line segment aligned with its central axis, while the KP method defines states based on key points positioned along each link. For the KP method, following the methodology in [22], we distribute key points along the central axis of each link at intervals equal to the link’s diameter (D). This heuristic is chosen to ensure sufficient volumetric coverage for collision detection without excessively increasing the state space dimension, while avoiding placement at the link ends to prevent artificial inflation of the robot’s volume. The OBB approach represents each link with an oriented bounding box that encloses its geometry. This formulation captures both the volumetric extent and orientation of the links, while reducing unnecessary computational overhead for simpler link geometries. All state representation structures are illustrated in Figure 12. All methods are trained using identical environmental settings and algorithmic configurations. In this comparison, the learning algorithm is fixed to MAPPO under the CTDE paradigm, so that any observed differences can be attributed solely to the choice of state representation. Building upon the reward formulation detailed in Equations (17) and (18), the KP method modifies computations by replacing line-segment distances with point-to-point distances, while the OBB method applies the same structure using approximated distances between bounding boxes. Each scenario is executed 100 times to assess its success rate, and convergence time is additionally measured during training to evaluate learning efficiency. Quantitative analysis results are presented in Table 9.

**Figure 13:**
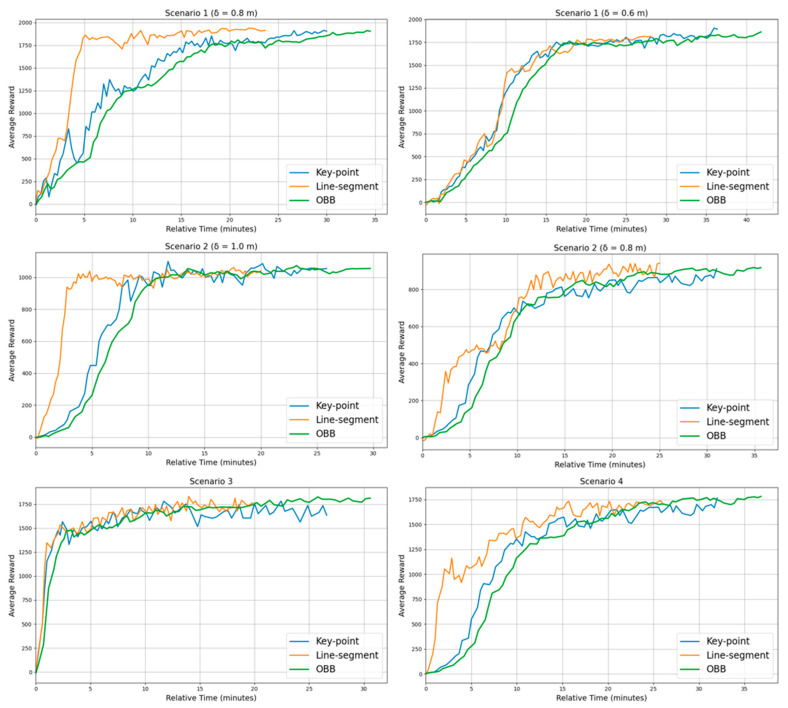
Average reward per relative time across all scenarios: line-segment (ours) vs. key point and oriented bounding box.

All state representation methods show a decline in the success rate and an increase in convergence time ($T_{conv.}$) as environmental complexity increases. In Scenario 1, all methods achieve similar success rates of about 93–94% and 88–89%, respectively. However, the LS method demonstrates a clear computational advantage, converging twice as fast as KP and significantly faster than OBB. In Scenario 2, LS and KP achieve comparable success rates (95–96%), while OBB records the highest value at 97%. Nevertheless, LS consistently outperforms both methods in convergence speed, requiring less than half the training time of KP and one-third that of OBB. Scenarios 3 and 4 evaluate environments with two and three agents, respectively. In Scenario 3, LS achieves a 97% success rate with the fastest convergence, surpassing KP and OBB. When the number of agents increases to three in Scenario 4, all methods exhibit lower success rates due to higher task complexity. Although KP and OBB slightly outperform LS in success rate (84% vs. 83%), LS maintains superior training efficiency, converging much faster. Figure 13 illustrates the average-reward learning curves, confirming the stable and efficient convergence of the LS method across all environments.

#### 3.3.4. Ablation Study on Avoidance Reward ($R_{avoid}$)

This subsection analyzes the contribution of the avoidance reward $R_{avoid}$ defined in Equation (17) through ablation experiments across different scenarios. Specifically, we compare learning performance when using only the collision penalty $R_{collision}$ and when additionally incorporating the avoidance reward.

As shown in Table 10 introducing $R_{avoid}$ consistently improves the success rate across scenarios. In Scenario 1, the success rate increases from 86.0% to 89.0% (+3.0%). In Scenario 2 the improvement is more pronounced, from 87.0% to 92.0% (+5.0%). In Scenario 4, where the environment includes higher collision risks, the success rate rises from 79.0% to 83.0% (+4.0%).

### 3.4. Performance Comparison in Task Environment

**Figure 14:**
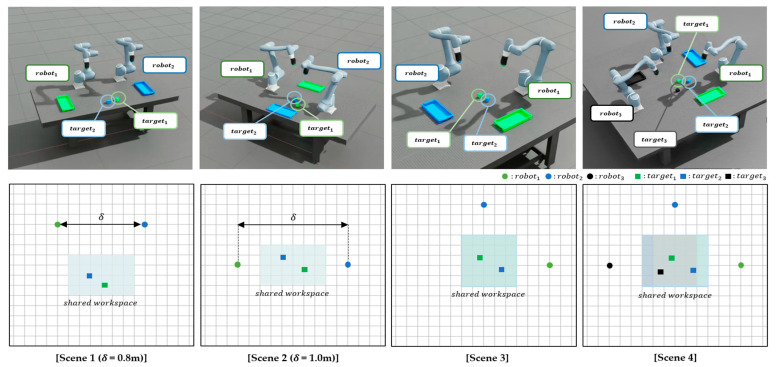
Four shared workspace (pick-and-place) scenes.

This section establishes four task simulation environments, illustrated in Figure 14, to validate the performance of the proposed method and compare it with the previously evaluated learning approaches discussed in Section 3.3.1 and Section 3.3.3. The experiments are conducted in environments consisting of two 6-DOF manipulators equipped with surface grippers, a set of cubes to transport, and their respective target boxes. The grid representations in Figure 14 display manipulator positions and cube-spawning regions. Manipulators are initialized in randomized starting states, and cubes are randomly generated within shared workspace regions at the beginning of each scene. Each manipulator is assigned a specific cube and performs a pick-and-place task by transferring the cube to a designated target box, with each cube and its corresponding box distinguished by color. Task execution time is defined as the duration required for both manipulators to successfully transfer their assigned cubes to the designated target boxes without collisions, with such collision-free transfers classified as successful task completions. To evaluate task success and execution efficiency, a total of 100 trials is conducted with randomized target configurations.

Table 11 summarizes the success rate (SR) and average time $T_{avg.}$ for tasks performed using previously trained configurations: line segment-based with independent value function (LS-Ind), line segment-based with centralized value function (LS-Cent), and key point-based with centralized value function (KP-Cent). Across all scenes, LS-Cent maintains consistently high performance with balanced SRs and the fastest $T_{avg.}$. In Scene 1 and Scene 2, LS-Cent achieves SRs of 90–93%, comparable to KP-Cent (91–93%), while reducing $T_{avg.}$ by nearly half (8–9 s vs. 14 s). Compared to LS-Ind (82–83%), LS-Cent exhibits notable improvements in both reliability and efficiency. In Scene 3, LS-Cent achieves the highest SR (95%), slightly surpassing KP-Cent (94%), and maintains the fastest $T_{avg.}$. Even in the more complex Scene 4, LS-Cent sustains competitive accuracy (83%)—close to KP-Cent (84%) and well above LS-Ind (62%)—while remaining faster (13 s vs. 17 s). Additionally, to evaluate the efficiency of the proposed multi-manipulator system, we compare its performance against a baseline emulating a conventional single-manipulator operation, in which multiple manipulators operate sequentially to avoid collisions. Table 12 presents the average task completion times obtained from 100 trials under randomized cube positions and robot initial positions for both the proposed simultaneous approach and the sequential baseline across the four scenarios.

**Figure 15:**
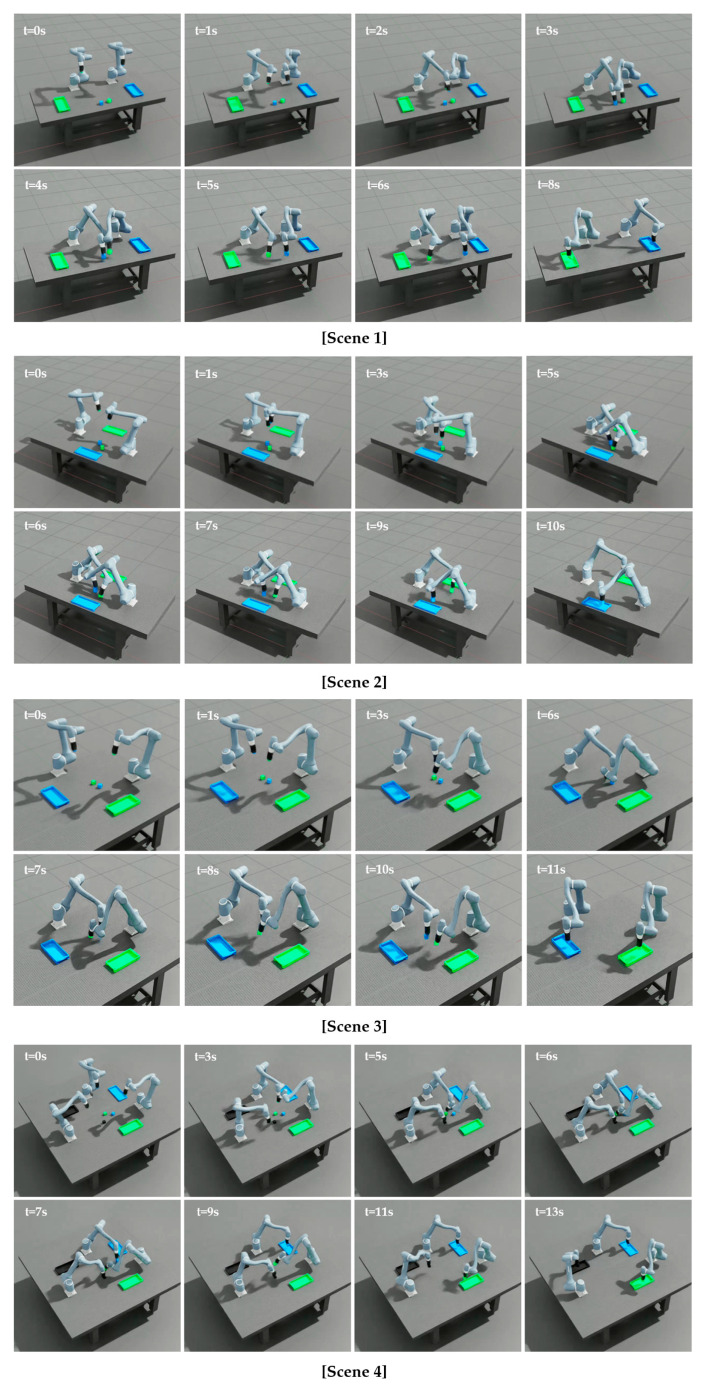
Visualization of successful cases across all (pick & place) scenes.

The multi-agent collaborative framework significantly reduces task completion times compared to the single-agent sequential baseline across all scenarios. As shown in Table 12, the average completion time decreases by approximately 50–60% in all scenes, with the most notable improvement observed in the dense workspace of Scene 4 (from 29 s to 13 s). This demonstrates that simultaneous multi-agent coordination greatly enhances overall efficiency without compromising task success. Figure 15 illustrates that the proposed method is effectively applied in the task environments, demonstrating successful task execution across all scenes.

### 3.5. Real-Time Performance Analysis

To verify whether the proposed RL-based navigation framework satisfies real-time operation requirements, we evaluate its computational performance across multiple test scenarios. Table 13 presents the FPS recorded over the last 100 steps in each scenario defined in Figure 14.

As shown in Table 13, all scenarios achieve an average FPS above 57, which corresponds to an update interval of approximately 18 ms or faster. This performance is more than sufficient to ensure stable, real-time motion planning, as the achieved frame rate comfortably exceeds the control frequency required for the robot to operate at its maximum joint velocities (180°/s for J1-J3 and 360°/s for J4-J6). This demonstrates that the learned policy can be executed with low latency, enabling smooth and reliable trajectory generation in real time.

### 3.6. Performance Comparison with Traditional Motion Planning Methods

**Figure 16:**
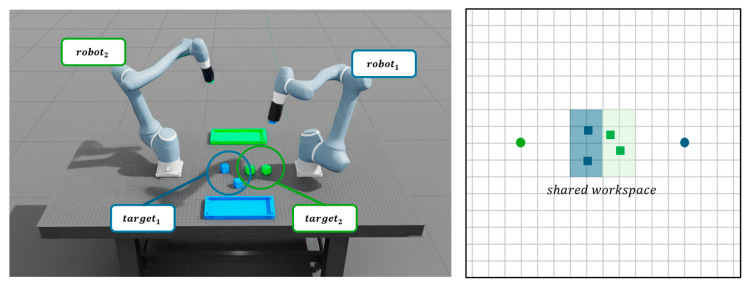
Workspace (pick-and-place) scene for benchmarking.

This section compares the proposed CTDE-based multi-agent reinforcement learning method with traditional motion planning approaches. The baseline planners include classical sampling-based methods integrated in OMPL—RRT-Connect [8], PRM [9], and PRM* [48]—as well as the optimization-based method CHOMP [13]. These planners have been widely adopted in both industry and academia and are considered standard benchmarks for manipulator motion planning. The experiments are conducted in a simulation environment built on NVIDIA Isaac Sim integrated with ROS2, using a two-manipulator setup where each manipulator is required to transport two cubes in the environment shown in Figure 16. Each task is repeated 20 times, and the results are summarized in the table with mean values and standard deviations. The evaluation compares execution time, average time $T_{avg.}$ for tasks performed, and success rate SR across these methods. Sampling-based planners guarantee probabilistic completeness, meaning that a solution can be found with nonzero probability within finite time. In all methods, trajectories are planned and executed simultaneously for both robots. If feasible trajectories cannot be found for both robots, one robot is reset to its initial state while the other completes the task. Table 14 summarizes the benchmark results in a setup with two manipulators.

- PRM [9] and RRT-Connect [8]: Average execution times are 46.98 s and 43.7 s, approximately twice that of MADRL (20.5 s). Path lengths (PRM: 4.87 m, 5.40 m; RRT-Connect: 4.13 m, 4.95 m) and success rates (75%, 72%) are also inferior to MADRL (2.95 m, 3.22 m; 92%). Although relatively faster among classical planners, both are less efficient and robust than MADRL.
- PRM* [48]: The success rate reaches 76%, the highest among sampling-based methods, with shorter paths (3.82 m, 3.86 m). However, its computation time of 205.7 s, which is more than ten times longer than that of MADRL, limits its practical applicability.
- CHOMP [13]: Average execution time is 177.8 s and success rate only 43%, with frequent collisions due to soft collision constraints. Although it generates smooth trajectories, CHOMP underperforms compared to MADRL in time efficiency and robustness.

The success rate of MADRL is 92%, measured as the proportion of trials in which both robots successfully perform pick-and-place simultaneously. This result demonstrates superior performance across nearly all metrics compared to the baseline planners. Moreover, MADRL offers the advantage of adaptability to dynamically changing environments. This flexibility contrasts with the baseline planners studied in this work, which are limited to predefined offline planning.

## 4. Discussion

Across diverse multi-manipulator settings, the method maintains high success. As spatial spacing tightens, success decreases but remains no lower than 89% in Scenarios 1 and 2. With increased agent density in Scenario 4, success declines to 83%, indicating that crowding is the primary limiting factor. To further assess performance under more challenging conditions, additional tests are conducted with four manipulators. In the four-manipulator environment, the proposed method achieves an 80% success rate, and the transition from three to four manipulators results in only a moderate decrease rather than a drastic drop. This represents a marginal decrease, suggesting that the proposed reward function and collision-detection mechanism maintain effectiveness even under more crowded conditions. Across all scenarios, tighter task constraints reduce success, but rates remain above 80%. Average return decreases slightly under higher collision risk (tighter spacing, greater density) yet stays stable overall, indicating robust and consistent policies. Convergence requires more updates in dense settings because elevated collision risk increases planning and coordination demands; residual return differences arise from structural factors such as robot placement, workspace overlap, target randomness, and initial poses.

When comparing performance with the independent value function (ind.) in multi-manipulator settings, the centralized value function (cent.) consistently outperforms it. This disparity intensifies in scenarios with more agents or shorter inter-manipulator distances, where interaction complexity and collision risk rise. In terms of convergence, the centralized value function generally reaches convergence with fewer training steps, indicating faster learning than its independent counterpart. These findings indicate that the cent-based learning structure facilitates more accurate and cooperative collision-avoidance strategies by incorporating all agents’ state and action information during training. The proposed framework is evaluated against representative CTDE algorithms—HAPPO [46], HATRPO [46], and HAA2C [47]—under identical training conditions (Figure 11). The results highlight that MAPPO achieves the most favorable balance of convergence speed and final performance, consistently reaching higher returns with fewer training steps. HAPPO exhibits performance that is nearly comparable to MAPPO [43] in most scenarios, suggesting that both methods are well suited for multi-manipulator coordination. MAPPO’s superior performance stems from its centralized critic, which leverages comprehensive global state information—including the positions, velocities, and intended actions of all agents—to provide more accurate value estimates during training. This richer contextual awareness reduces the variance of policy-gradient updates and improves credit assignment across agents, thereby accelerating convergence and enabling the learning of more coordinated behaviors. These results reinforce the suitability of MAPPO as the backbone of the proposed framework. Its ability to efficiently incorporate shared state information and optimize cooperative behaviors enables it to outperform other CTDE variants, particularly in scenarios with high agent density and complex collision risks. At the same time, the comparable performance of HAPPO suggests that alternative CTDE-based methods can also serve as viable extensions, depending on specific stability or scalability requirements.

The LS (line-based) representation achieves a success rate comparable to the KP (point-based) method [22], while consistently converging faster across all scenarios. In contrast, the OBB (bounding-box-based) approach provides the most detailed volumetric modeling and achieves the highest success rate but requires significantly more computation. Overall, LS offers the best trade-off between accuracy, convergence speed, and computational efficiency, making it the most practical choice for multi-manipulator reinforcement learning. Moreover, its computational efficiency makes the line-based method ideal for embedded systems with constrained GPU or CPU resources. Consequently, the LS representation serves as a state representation technique that provides scalability and practicality for reinforcement learning-based manipulator control.

In the task simulation environment, the proposed LS-Cent approach demonstrates consistent and balanced performance across all scenes. The KP-Cent method achieves a similarly high success rate but shows the longest completion time (14–15 s) due to its higher computational complexity. In contrast, LS-Ind completes tasks the fastest but records the lowest success rate (82%), highlighting the limitation of independent learning, where the absence of inter-agent information sharing reduces stability and reliability in complex multi-manipulator environments. A slight performance variance is observed in complex scenarios, where the KP method achieves marginally higher success rates than the LS method. This difference arises from a trade-off in representational fidelity: the LS approach abstracts links into line segments for efficiency, which does not fully capture the link volume, while KP more accurately models geometry, including extremities and joints. In dense environments, this fidelity gives KP a minor advantage in collision detection sensitivity. However, the marginal gain in success rate comes with a significant increase in completion time, highlighting the overall efficiency of the LS approach. In the same settings, single-manipulator sequential operations maintain task stability but show significant limitations in task efficiency, with average task times exceeding 18 s across all scenarios. Although multi-manipulator cooperative policies have slightly lower stability compared to single-manipulator operations, they significantly enhance task efficiency and productivity, reducing completion times to less than half while maintaining acceptable success rates. These results experimentally validate that the proposed line-based state representation, combined with a centralized value function learning structure, forms an effective learning framework for collision avoidance and path planning in multi-manipulator collaborative environments. In addition, the runtime analysis shows that all scenarios achieve average frame rates above 57 FPS, corresponding to update intervals of about 18 ms or faster. This confirms that the learned policies can be executed with low latency, enabling smooth and reliable real-time motion planning even under increased manipulator density.

However, several limitations of the proposed method should be acknowledged. First, the scalability of the proposed framework is not yet fully validated. Although it extends to four manipulators, the success rate decreases from over 90% with two manipulators to about 80%, indicating the need for further improvement to ensure reliable scalability for larger robot teams. In addition, the failure case analysis shows that as environments become denser, collisions emerge as the dominant cause of failures. Based on the analysis, the primary cause of these failures is attributed to the limitations of the state representation. As the number of manipulators increases and the workspace becomes more densely populated, the proposed line-segment-based state representation sacrifices geometric fidelity to maintain computational efficiency. Consequently, it fails to accurately capture the complex interactions between closely spaced links and struggles to precisely detect collision scenarios. These representational limitations are identified as the main factors contributing to performance degradation and task failures in high-density environments. Although alternative methods such as point-based or bounding-box-based representations offer higher geometric accuracy, their computational complexity grows exponentially with the number of manipulators, making them unsuitable for large-scale systems. Consequently, the line-segment-based state representation remains the main limiting factor for reliable collision avoidance in densely populated multi-manipulator environments.

Second, in this study, the manipulator’s end-effector (EE) orientation is not explicitly included in the state representation or policy learning. While sufficient for demonstrating collision-free position control, this simplification may reduce precision in tasks requiring accurate EE orientation. Preliminary experiments with an added orientation-alignment reward show that the framework maintains stable collision avoidance while learning EE posture control, confirming its potential for extension to more complex, high-dexterity tasks. Third, the proposed line-segment-based state representation inherently assumes that manipulator links can be approximated as cylindrical shapes. While this simplification is effective for standard link geometries, it may not accurately capture non-cylindrical links or more complex end-effectors, such as multi-fingered grippers or specialized tools. This limitation could reduce modeling fidelity in tasks where the detailed geometry of the manipulator plays a critical role.

In future research, enhancing the scalability of the proposed framework is essential to ensure stable performance as the number and diversity of manipulators increase. While the current method maintains over 80% success rates in simulation, it still faces stability limitations when applied to real industrial environments. As more robots operate within a shared workspace or as manipulator structures become more complex, its performance may degrade due to increased interaction dynamics. The line-segment-based state representation simplifies link geometry for computational efficiency; however, it shows clear limitations in accurately reflecting nonlinear structures, complex link shapes, and the overall geometric configuration of manipulators. To overcome these limitations, future work can adopt a hybrid modeling strategy in which standard cylindrical links are represented as line segments, while complex or irregular components are approximated with additional geometric primitives such as points or spheres. This extension will allow the framework to adapt to manipulators of various shapes and dexterities, improving both scalability and generalization for real-world manipulator systems. Improvements in these areas are expected to yield more robust learning, higher reliability, and better adaptability for deployment in real-world multi-manipulator environments. Building on this requirement, the primary focus of future research should be on applying the proposed framework in real-world environments.

All experiments and evaluations were exclusively conducted within the NVIDIA Isaac Sim simulation environment without deployment on actual robotic hardware. Given that reinforcement learning-based policies often suffer performance degradation owing to the Sim-to-Real gap, additional validation in real-world settings is crucial to verify the generalization and applicability of the method. Recent studies have shown promising directions to mitigate the sim-to-real gap, such as structured and adaptive domain randomization (e.g., GoFlow) [49] and real-to-sim-to-real refinement with digital twin calibration [50]. Building upon these advances, our future research will also expand in these directions to enhance the transferability and robustness of the proposed method. In industrial applications, manipulators often handle workpieces of varying weights, where load variations directly affect torque, accuracy, and response speed. However, our experiments were conducted under fixed or no-load conditions, without measuring critical metrics such as motor energy consumption. While the line-segment representation accelerates convergence, it may also induce more complex trajectories, potentially increasing energy usage. Moreover, the experimental scenarios considered in this study were static and structured, with fixed targets, no dynamic obstacles, and limited randomness in initial postures. Such simplifications limit realism and fail to capture the dynamic and uncertain nature of industrial environments. Future work should therefore incorporate practical constraints such as varying loads, energy consumption, and dynamic task conditions to more faithfully assess the applicability of the proposed framework in real-world settings. In addition, real industrial applications often require manipulators to collaborate not only with other robots but also with humans in shared workspaces. Recent advances in human–robot interaction and sensing, such as muscle–computer interfaces for hand gesture recognition using depth vision [51] and AIoT-based human activity recognition frameworks [52], highlight promising directions. Incorporating such multimodal sensor data into the learning process could enhance adaptability and robustness when extending the proposed approach to practical deployment.

For the deployment of reinforcement learning in real-world industrial environments, it is essential to address advanced control objectives focused on safety and robustness. As part of recent reinforcement learning achievements related to these safety issues, integrating fault-tolerant control mechanisms that ensure safe operation even under system faults [24], or applying robust control strategies that maintain resource efficiency under external disturbances [25], can enable effective responses to unpredictable situations. In addition, recent studies such as self-triggered approximate optimal neuro-control for nonlinear systems [23], event-triggered secure tracking control [53], and observer-based fault-tolerant control [54] have all advanced reinforcement learning-based control techniques toward guaranteeing safe operation by considering various uncertainties and fault conditions that may arise in real-world scenarios. Recent perception-driven research such as the lightweight attention-guided network for underwater object detection [55] demonstrates the effectiveness of integrating attention-based and cross-scale feature interactions to improve environmental perception under complex visual conditions. Incorporating such perception modules into multi-agent reinforcement learning frameworks could further enhance spatial awareness, robustness, and adaptability in unstructured or dynamic environments. Furthermore, recent advances in robust reinforcement learning under adversarial attacks, such as the studies by Ziyuan Zhou et al. [56,57] demonstrate that multi-agent deep reinforcement learning (MADRL) systems are vulnerable to malicious perturbations in states or actions. Their research analyzes how adversarial disturbances can degrade cooperative policy performance and proposes defense mechanisms that enhance the robustness and stability of learned policies against such attacks. Considering these trends, our future work will focus on identifying potential safety challenges in real-world applications of RL-based control and developing strategies to address them to ensure reliable and safe deployment.

Additionally, in this study, the proposed framework has primarily focused on accuracy and speed, while energy efficiency has not been sufficiently considered, which may lead to excessive energy consumption and increased mechanical stress during operation. Therefore, incorporating energy-aware control strategies into the proposed framework is an essential future direction. In particular, adopting approaches such as the robust and energy-efficient trajectory planning framework proposed by Hussain et al. [58] could further enhance the system’s performance by balancing precision, robustness, and energy consumption.

## 5. Conclusions

This study introduces a MADRL framework for collision avoidance and motion planning in a multi-manipulator system, where manipulators operate within SWEs. To enable collision avoidance through inter-manipulator state awareness, manipulator link states are represented by line segments, thereby enhancing the expressiveness of the state space. Additionally, a reward structure based on link distances is developed to ensure both computational efficiency and effective collision avoidance. Experimental results indicate that the proposed line-based state representation outperforms the traditional state representation approach in terms of convergence time and computational efficiency, making it well-suited for multi-manipulator collaborative settings.

A centralized value function structure is utilized, incorporating a global state that combines the relative positions and actions of all agents. This architecture enables more accurate value estimation by considering not only each manipulator’s own state but also the dynamic states of neighboring manipulators, thereby facilitating more effective policy learning. The experimental results indicate that, compared to an independent value function (ind.) structure, cent. achieves superior performance in collision-sensitive environments. These findings demonstrate that the proposed approach effectively prevents inter-manipulator collisions while promoting stable policy convergence.

In conclusion, the proposed method executes tasks safely and reliably within collaborative multi-manipulator settings, highlighting its strong potential for real-world deployment. While further improvements are still required for practical deployment in environments involving three or more manipulators, the findings nevertheless indicate a clear direction and promising applicability of the proposed approach to such challenging scenarios. Furthermore, this study advances the development of safe and efficient multi-manipulator systems and lays the groundwork for future research on enhanced state representations, benchmarking against alternative planning algorithms and validating real-world robotic applications.

## Figures and Tables

**Table 1:** Hyperparameters used in MAPPO across all domains.

Common Hyperparameters	Value
recurrent data chunk length	10
gradient clip norm	1.0
gae lambda	0.95
gamma	0.99
value loss	huber loss
batch size	num envs × buffer length × num agents
mini batch	16
optimizer	Adam
optimizer epsilon	1 × 10^−5^
weight decay	0

**Table 2:** Explanation of evaluation metrics.

Metrics	Explanation
SR	Success Rate: Percentage of episodes in which the manipulator successfully reaches the target without collisions or timeouts.
FR	Failure Rate: Percentage of episodes in which the manipulator failed due to collisions or timeouts.
AR	Average Reward: Mean and standard deviation of cumulative rewards across episodes, indicating overall policy performance.
CS	Convergence Step: The training step at which the smoothed reward first reaches 95% of the final reward value.
$T_{conv.}$	Convergence Time: Time (in minutes) required for the training to reach stable policy performance.
$T_{avg.}$	Average Time: Average time required for all manipulators to complete their tasks.

**Table 3:** Success rates for safety margin (ϵ) tuning in various scenarios.

Scenario		SR (%)				
		ϵ = 0.01	ϵ = 0.03	ϵ = 0.05	ϵ = 0.08	ϵ = 0.1
Scenario 1	*δ* = 0.8 m	72.0	86.0	93.0	88.0	80.0
	*δ* = 0.6 m	68.0	80.0	89.0	83.0	75.0
Scenario 2	*δ* = 1.0 m	79.0	89.0	95.0	91.0	84.0
	*δ* = 0.8 m	74.0	85.0	92.0	87.0	81.0
Scenario 3		83.0	91.0	97.0	93.0	87.0
Scenario 4		64.0	75.0	83.0	78.0	70.0

**Table 4:** Success rates under varying reward coefficients (ω) in Scenario 2.

Scenario 2	Reward Coefficients			SR [%]
	$\omega_{dist}$	$\omega_{action\_penalty}$	$\omega_{avoid,t}$	
*δ* = 1.2 m	1.0	−0.001	5.0	97.0
*δ* = 1.0 m	1.0	−0.001	5.0	93.0
	0.5	−0.001	5.0	90.0
	1.0	−0.0001	5.0	95.0
	1.0	−0.0001	10.0	92.0
	1.5	−0.0001	5.0	88.0
*δ* = 0.8 m	1.0	−0.0001	5.0	92.0
	0.5	−0.0001	5.0	88.0
	1.0	−0.0001	10.0	90.0
	1.5	−0.0001	10.0	87.0

**Table 5:** Performance of the proposed method in various scenarios.

Scenario		SR (%)	FR (%)		AR	CS
			Collision	Timeout		
Scenario 1	*δ* = 0.8 m	93.0	4.0	3.0	1923.16 ± 3.23	10,800
	*δ* = 0.6 m	89.0	7.0	4.0	1798.64 ± 17.39	26,640
Scenario 2	*δ* = 1.0 m	95.0	3.0	2.0	1032.18 ± 7.80	7920
	*δ* = 0.8 m	92.0	5.0	3.0	940.91 ± 7.71	19,080
Scenario 3		97.0	2.0	1.0	1742.51 ± 14.74	14,760
Scenario 4		83.0	12.0	5.0	1565.18 ± 4.79	20,160

**Table 6:** Performance of the proposed method in four-manipulator environment.

Scenario	SR (%)	FR (%)		AR	CS
		Collision	Timeout		
Four manipulators	80.0	16.0	4.0	1485.38 ± 15.79	23,160

**Table 7:** Quantitative comparison of centralized value function ($V_{\varphi }$) and independent value function ($V_{\varphi }$).

Scenario		SR (%)		CS	
		$\mathrm{Ind}.\;V_{\varphi }$	$\mathrm{Cent}.\;V_{\varphi }$ (Ours)	$\mathrm{Ind}.\;V_{\varphi }$	$\mathrm{Cent}.\;V_{\varphi }$ (Ours)
Scenario 1	*δ* = 0.8 m	88.0	93.0	19,940	10,800
	*δ* = 0.6 m	76.0	89.0	28,080	25,040
Scenario 2	*δ* = 1.0 m	89.0	95.0	13,000	7920
	*δ* = 0.8 m	77.0	92.0	29,120	19,080
Scenario 3		82.0	97.0	26,070	14,760
Scenario 4		66.0	83.0	23,280	20,160

**Table 8:** Quantitative comparison of policies across various environments used for HAPPO, HATRPO, HAA2C, and MAPPO (ours).

Scenario		SR (%)			
		HAPPO	HAA2C	HATRPO	MAPPO (Ours)
Scenario 1	*δ* = 0.8 m	93.0	84.0	92.0	93.0
Scenario 2	*δ* = 1.0 m	94.0	83.0	93.0	95.0
Scenario 3		97.0	90.0	91.0	97.0
Scenario 4		82.0	76.0	78.0	83.0

**Table 9:** Quantitative comparison of state representation methods.

Scenario		SR (%)			$T_{conv.}$(min)		
		KP	OBB	LS (Ours)	KP	OBB	LS (Ours)
Scenario 1	*δ* = 0.8 m	93.0	94.0	93.0	18.55	22.32	9.76
	*δ* = 0.6 m	88.0	89.0	89.0	26.28	31.22	19.81
Scenario 2	*δ* = 1.0 m	96.0	97.0	95.0	12.02	15.82	5.37
	*δ* = 0.8 m	92.0	94.0	92.0	21.02	24.25	13.15
Scenario 3		96.0	96.0	97.0	18.54	21.32	10.79
Scenario 4		84.0	84.0	83.0	28.42	34.67	20.32

**Table 10:** Quantitative results of the ablation study on the avoidance reward across various scenarios.

Scenario		Method	SR (%)
Scenario 1	*δ* = 0.6 m	$R_{collision}$	86.0
		$R_{collision}$ + $R_{avoid}$	89.0
Scenario 2	*δ* = 0.8 m	$R_{collision}$	87.0
		$R_{collision}$ + $R_{avoid}$	92.0
Scenario 4		$R_{collision}$	79.0
		$R_{collision}$ + $R_{avoid}$	83.0

**Table 11:** Quantitative comparison of policies across various environments.

	SR (%)			$T_{avg.}$(s)		
Scene	LS-Ind	KP-Cent	LS-Cent (Ours)	LS-Ind	KP-Cent	LS-Cent (Ours)
Scene 1	83.0	91.0	90.0	9	14	10
Scene 2	82.0	93.0	93.0	8	15	9
Scene 3	81.0	94.0	95.0	10	17	10
Scene 4	62.0	84.0	83.0	11	16	14

**Table 12:** Quantitative comparison of single and multi-manipulator task performance.

	$T_{avg.}$(s)			
Scene	Scene 1	Scene 2	Scene 3	Scene 4
Single	19	17	22	30
Multi (Ours)	10	9	10	14

**Table 13:** Runtime performance summary across different scenes.

	Scenes			
	Scene 1	Scene 2	Scene 3	Scene 4
Avg. FPS	65.88	64.44	59.99	57.95

**Table 14:** Benchmark results for 20 tasks in a setup with 2 manipulators.

Method	$T_{avg.}$(s)	L(m)		SR (%)
		${Robot}_{1}$	${Robot}_{2}$	
PRM [9]	46.98 ± 8.7	4.87 ± 1.64	5.40 ± 1.93	75
PRM* [48]	205.7 ± 37.7	3.82 ± 0.98	3.86 ± 1.03	76
RRT-Connect [8]	43.7 ± 8.1	4.13 ± 1.32	4.95 ± 1.84	72
CHOMP [13]	177.8 ± 37.5	3.29 ± 0.69	4.47 ± 1.31	43
MADRL (ours)	20.5 ± 5.8	2.95 ± 0.92	3.22 ± 1.13	92

## Data Availability

The original contributions presented in this study are included in the article/Supplementary Material. Further inquiries can be directed to the corresponding author.

## Supplementary Materials

The following supporting information can be downloaded at: https://www.mdpi.com/article/10.3390/s25226822/s1, Video S1: Demonstration of multi-robot manipulation.
